# Assessing the perceived impact of post Minamata amalgam phase down on oral health inequalities: a mixed-methods investigation

**DOI:** 10.1186/s12913-019-4835-1

**Published:** 2019-12-21

**Authors:** V. R. Aggarwal, S. Pavitt, J. Wu, B. Nattress, P. Franklin, J. Owen, D. Wood, K. Vinall-Collier

**Affiliations:** School of Dentistry, Faculty of Medicine & Health, University of Leeds, Worsley Building, Clarendon Way, Leeds, LS2 9LU UK

**Keywords:** Amalgam, Phase-down, Minamata, Inequalities, Oral health

## Abstract

**Background:**

Data from countries that have implemented a complete phase out of dental amalgam following the Minamata agreement suggest increased costs and time related to the placement of alternatives with consumers absorbing the additional costs. This aim of this study was to investigate the impact of a complete phase out of dental amalgam on oral health inequalities in particular for countries dependent on state run oral health services.

**Methods:**

A mixed methods component design quantitative and qualitative study in the United Kingdom. The quantitative study involved acquisition and analysis of datasets from NHS Scotland to compare trends in placement of dental amalgam and a survey of GDPs in Yorkshire, UK. The qualitative study involved analysis of the free text of the survey and a supplementary secondary analysis of semi-structured interviews and focus groups with GDPs (private and NHS), dental school teaching leads and NHS dental commissioners to understand the impact of amalgam phase down on oral health inequalities.

**Results:**

Time-trends for amalgam placement showed that there was a significant (*p* < 0.05) reduction in amalgam use compared with composites and glass ionomers. However dental amalgam still represented a large proportion (42%) of the restorations (circa 1.8 million) placed in the 2016–2017 financial year.

Survey respondents suggest that direct impacts of a phase down were related to increased costs and time to place alternative restorations and reduced quality of care. This in turn would lead to increased tooth extractions, reduced access to care and privatisation of dental services with the greatest impact on deprived populations.

**Conclusion:**

Amalgam is still a widely placed material in state run oral health services. The complete phase down of dental amalgam poses a threat to such services and threatens to widen oral health inequalities. Our data suggest that a complete phase out is not currently feasible unless appropriate measures are in place to ensure cheaper, long-lasting and easy to use alternatives are available and can be readily adopted by primary care oral health providers.

## Background

In October 2013 a Global treaty (Minamata 2013) [1] agreed to phase out the use of dental amalgam as there was sufficient evidence of significant global adverse impact from mercury and its compounds to warrant international action to reduce these risks [1]. Over several years this was worked on with international governments and the need for a binding agreement was found. The Minamata Convention was agreed in January 2013 and has the objective to “protect the human health and the environment from anthropogenic emissions and releases of mercury and mercury compounds” [1]. The convention contains measures to meet this objective and key objectives pertinent to dental amalgam use were:

- Setting national objectives aimed at minimizing (amalgam) use;
- Promoting the use of cost-effective and clinically-effective mercury-free alternatives;
- Promoting research and development of quality mercury-free materials for dental restoration;
- Encouraging professional societies and dental schools to educate and train dental professionals in the use of mercury-free dental restoration;
- Encouraging insurance policies and programs that favor the use of quality alternatives to amalgam;
- Promote the use of best environmental practices in dental facilities to reduce releases of mercury and mercury compounds to water and land.

However, the World Health Organisation, World Dental Federation (FDI), International Association for Dental Research (IADR) and UK organizations like the British Dental Association (BDA) lobbied for the gradual reduction in the use of dental amalgam rather than an unworkable complete ban [2].

They argued that a total ban could ‘destabilize dentistry globally and time was needed to enable a phase down, as well as the need to develop comparable alternative materials for restorations. A true alternative to amalgam that could be operationalised in dentistry has yet to be established and, until then, it is accepted that a ban would risk adversely affecting public health and destabilize an already complex service delivery process’ [2].

Countries such as Norway and Sweden have implemented the phase-out process with a complete ban in the use of dental amalgam in 2008 and 2009 respectively. A review of the post-ban experiences in Norway suggest that although the experiences with alternative materials are positive, they are more time consuming and technique sensitive to use and impose increased costs to patients and providers [3]. The useful life of an amalgam restoration for a permanent posterior tooth was longer compared with a composite resin restoration (amalgam: 132.6 months versus composite: 95.7 months) and costs less (amalgam: $171 versus composite: $219) [4]. Time-to-failure was longer for amalgam restorations, resulting in a lifetime cost that was estimated to be half that of composite resin restorations when assuming that a failed restoration would be replaced by another of the same size and of the same material [4]. This raises particular concerns in countries where alternatives are unaffordable within their state run health services. Countries that are vulnerable include the United Kingdom where under the current National Health Service (NHS) dental contract (revised in 2006) dentists operate in an incentivized payment system. The NHS reimbursement process is akin to health insurance systems in other parts of the globe that provide cheaper alternatives for access to oral health services. Amalgam placements offer an operational solution for reimbursement that is typically on the lowest payment tier; amalgams can be placed relatively quickly; typically in half or a third of the time to composite materials and offer good longevity.

Composites and other mechanistically distinct alternative materials are routinely used in private practice - to the almost exclusion of amalgam. This successful adoption and diffusion can largely be explained by this sector not being limited by the same reimbursement constraints. However it remains highly improbable that these alternative materials to amalgam with their typical significant time burden of placement, could offer affordable tangible alternatives to support adoption in for example state run oral health services like the NHS in their current guise.

A deeper understanding of stakeholders is needed in particular of barriers and enablers for: [1] workforce (GDPs) (including their educational need and their financial perspectives) [2]; policy makers’ (commissioners) requirements to develop evidence informed models for the phase-down of amalgam. Whilst Minamata is driving the urgency for amalgam phase down, it potentially threatens to widen oral health inequalities given the wide use of amalgam within oral health services that offer cheaper access to oral health care.

The primary aim of this study was therefore to understand the perceived impact of amalgam phase down on oral health inequalities by exploring key stakeholder perspectives; notably General Dental Practitioners (GDPs) considerate of operational and educational needs that address their perceived barriers around using amalgam and its alternatives; the requirement/expectations of commissioners in the current and evolving dental contract reform era. This will inform the likely consequences of an amalgam phase down that may be addressed in a timely fashion by, for example, evaluation of newer cost-effective materials and/ or development of improved pragmatic protocols for the smarter use of the existing vast array of non-amalgam materials.

Specific Objectives:

1. To interrogate United Kingdom (UK) general dental practice data to explore the progress of a gradual reduction in the use of dental amalgam.
2. To explore the perceptions of GDPs, dental educators and dental commissioners on the impact of amalgam phase down on oral health inequalities

## Methods

A mixed methods component design study was conducted. The quantitative phase assessed trends in the use of amalgam in primary dental care to examine amalgam phase down status. The qualitative phase involved analysis of responses to open ended questions of a survey of Yorkshire dentists and also supplementary analysis of semi-structured interviews and focus groups with stakeholders (GDPs, Dental School leads and commissioners). Supplementary analysis here is in keeping with that described by Heaton J [5] as “a more in depth analysis of an emergent issue that was only partially addressed in the primary study” [5]. The objective of the primary study was to explore the specific needs of these groups and their patients to move towards amalgam free practice. Supplementary analysis here will address the impact of amalgam phase down on oral health inequalities.

### Quantitative phase

We used the National Health Service General Dental Service (GDS) data in Scotland to determine the trends in amalgam use for over 10 years up to 2017. The NHS GDS is the first point of contact for NHS dental treatment. Data is extracted from MIDAS (Management Information & Dental Accounting System), the computerised payment system for dentists providing GDS. The items of service (i.e. treatments) that dentists can provide and claim payment for are listed in the Statement of Dental Remuneration (SDR) [6].

We calculated the number of treatments for the following items taken from the Statement of Dental Remuneration to represent the number of fillings placed for each type of restoration [6]:

- 14(a) & 58(b) - Amalgam fillings in permanent or retained deciduous teeth
- 14(c,1) & 58(c,1) - composite resin or synthetic resin filling, including acid etch retention
- 14(c,2) & 58(c,2) - glass ionomer, silicate or silico-phosphate filling

### Quantitative survey of dental practitioners

A questionnaire was developed and distributed by post to 350 registered dentists in the West Yorkshire area selected at random from the UK Dentists register. The questions requested information on the factors that could affect a phase down in amalgam including awareness of information, current practice, concerns with alternatives and whether amalgam is still seen as a useful material. Both closed and open ended questions were used and responses to both were analysed for the quantitative and qualitative analyses respectively. The survey included both NHS and private dentists. Full details of the questionnaire and survey are available in Additional file 1 (supplementary attachment).

### Analysis including stratification by awareness of phase down

We undertook descriptive analysis of the responses examining awareness of a phase-down, current practice for choice of restorative materials and concerns with alternatives and whether amalgam is a useful material. To account differences by knowledge of a phase-down, we stratified the analysis by responses to the question ‘Are you aware of any publications or information regarding a reduction in the amount of amalgam that is used in the UK?’ (Question 6 Additional file 1).

### Qualitative phase

Informed consent was obtained from all participants prior to participations. Focus groups and interviews were undertaking using a topic guide (Additional file 2) for the researchers to prompt with open-ended questioning, allowing for exploration of issues generated by the participant. The interviewers adopted a flexible framework for questioning, using open questions to elicit data from participants and probing to focus on relevant details. The topic guide (Additional file 2) was developed following a review of the literature and informed by 3 focus groups: (GDPs [Focus Gr 1] newly trained and [Focus Gr 2] experienced GDPs) and with dental therapists [Focus Gr 3] and was further developed in parallel with the analysis using a constant comparative technique (cycling between data and analysis). Focus groups 1 and 3 were led by the social scientist and focus group 2 by the dentist. Focus group 1 lasted 25 min, focus group 2 lasted 27 min and focus group 3 lasted 29 min. Topics covered experiences and understanding of amalgam phase out, barriers to use of alternatives to amalgam and educational needs to facilitate NHS adoption and diffusion.

### Sample and procedure

A sample of GDPs, Dental School Leads and Commissioners were recruited via convenience and snowball sampling methods from organizations throughout the UK with GDPs centering in the North of England. GDP participants were purposively sought to provide maximum variance in range of views (newly trained & experienced GDPs, NHS and Private). Where necessary deviant cases were sought to test emerging hypotheses (notably a community dentist, foundation dentist and hygiene and therapists). Data collection and analysis continued until thematic saturation was achieved [7].

Data were collected by two researchers; one social scientist and one dentist. Focus groups with GDPs and hygienists and therapists working within the hospital setting took place on University of Leeds premises. Written consent was gained for all interviews and consent to publish anonymised quotes. Individual interviews took place over the telephone or face to face depending on preference of the participant. All were recorded and transcribed verbatim and lasted between 15 and 70 min. Data was analysed for emerging themes and categories identified.

Primary analysis of the data was undertaken by one author (KVC) and a deductive thematic analysis was carried out in line with recommendations [8, 9]. This resulted in four overarching themes to answer the research question of what are the specific needs of GDPs, dental school leads, commissioners and patients to move towards amalgam free practice. Secondary analysis of this data set was primarily undertaken by the second author VA in collaboration with KVC to re-examine the data set to look more in detail at the impact of amalgam phase down on oral health inequalities. The results from the free-text answers of the quantitative survey of Yorkshire dentists were analysed using content analysis and combined with the results of the secondary analysis to investigate the impact of amalgam phase down on oral health inequalities.

## Results

### Current use of amalgam and alternatives

To meet objective 1: To interrogate United Kingdom (UK) general dental practice data to explore the progress of a gradual reduction in the use of dental amalgam.

**Fig. 1:**
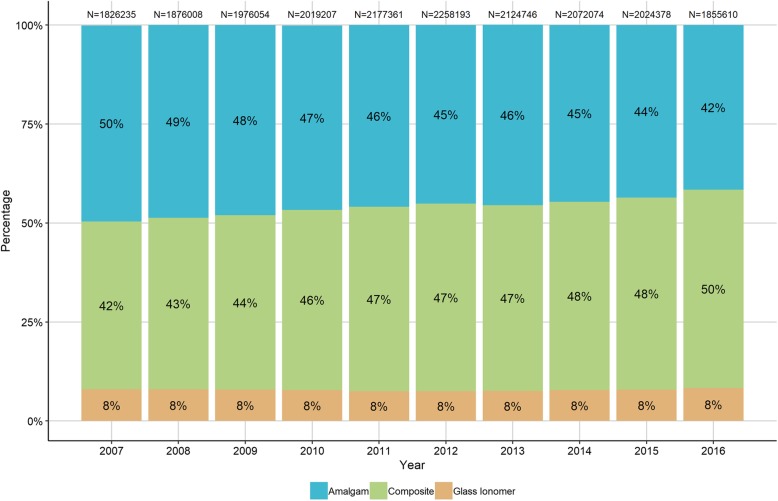
Types of filling materials used in Scotland **–** data from NHS Scotland showing the relative percentages of amalgams, composites and glass ionomer fillings placed in the NHS over a 10 year period between 2007 and 2016

**Fig. 2:**
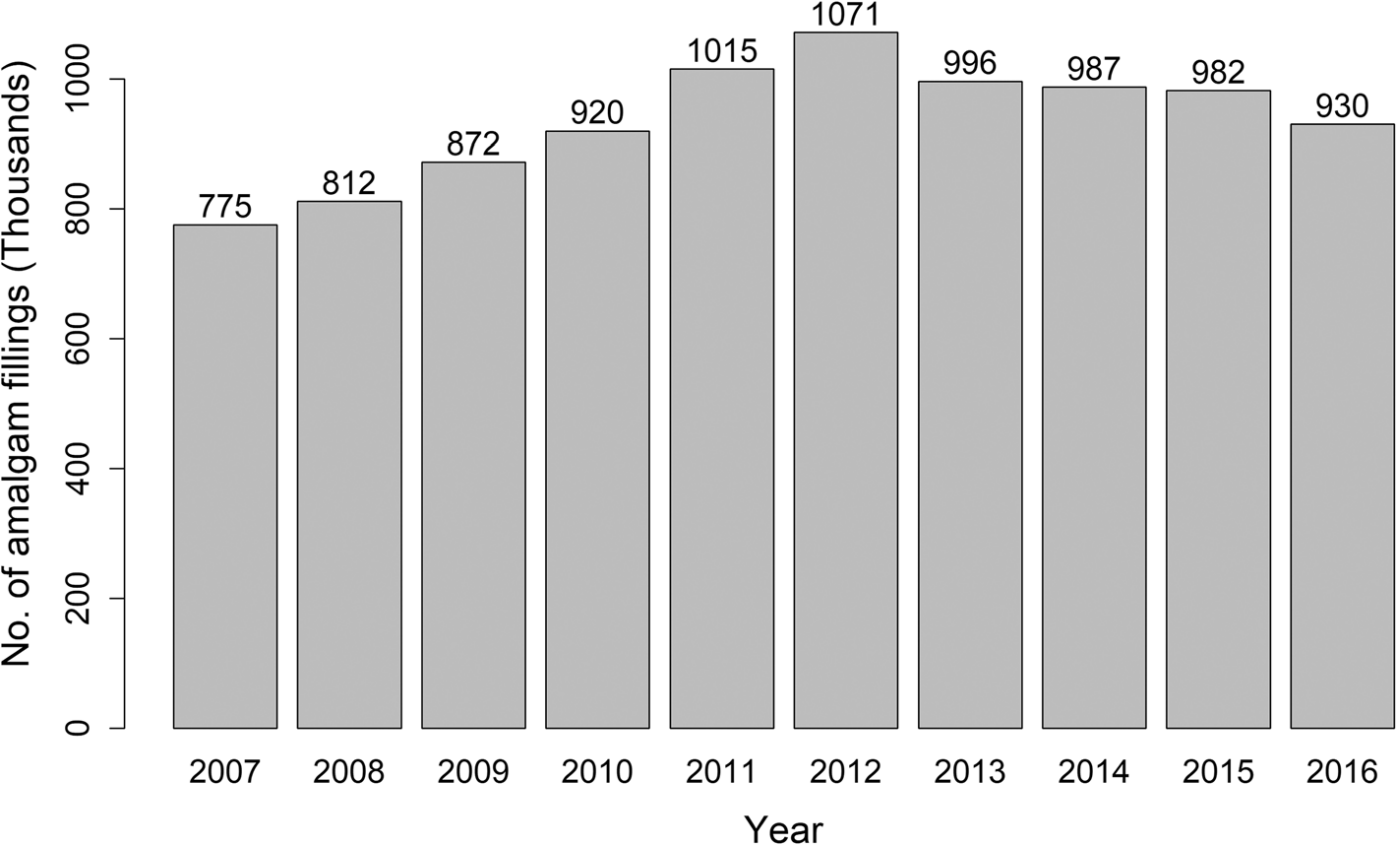
The usage of amalgam fillings in Scotland **–** data from NHS Scotland showing trends in amalgam fillings placed on the NHS over a 10 year period between 2007 and 2016

Time-trends for amalgam placement showed that there was a significant (*p* < 0.05) reduction in amalgam use compared with composites and glass ionomers (Fig. 1). However dental amalgam still represented a large proportion (42%) of the restorations (circa 930 thousand) placed in the 2016–2017 financial year (Fig. 2).

**Fig. 3:**
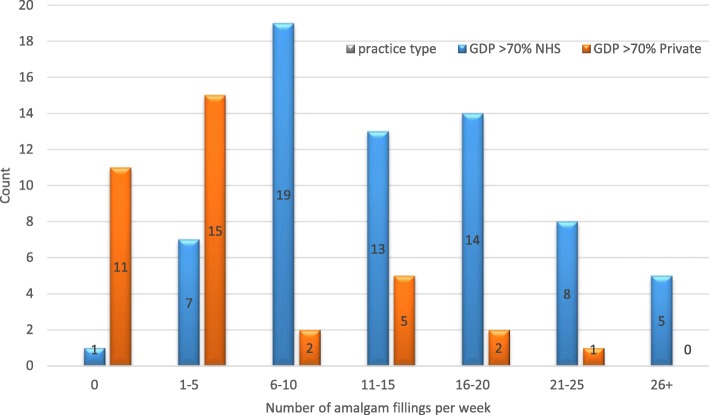
Amalgam usage by practice **–** data from the Yorkshire survey of dentists comparing the number of amalgam fillings placed per week between predominantly NHS (> 70% NHS) and predominantly private (> 70% private) practices

**Fig. 4:**
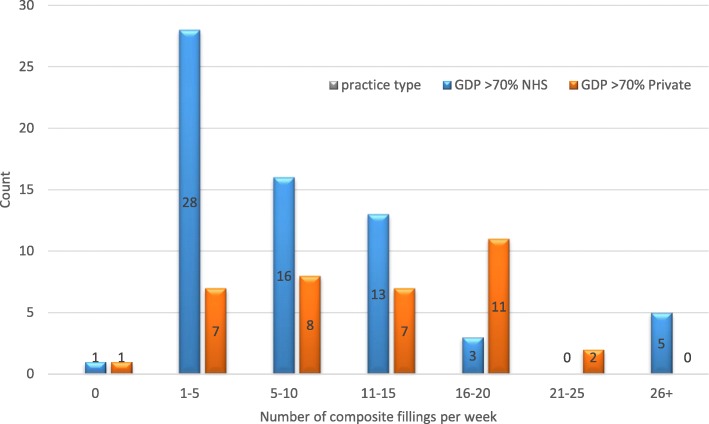
composite usage by practice **-** data from the Yorkshire survey of dentists comparing the number of amalgam fillings placed per week between predominantly NHS (> 70% NHS) and predominantly private (> 70% private) practices

**Fig. 5:**
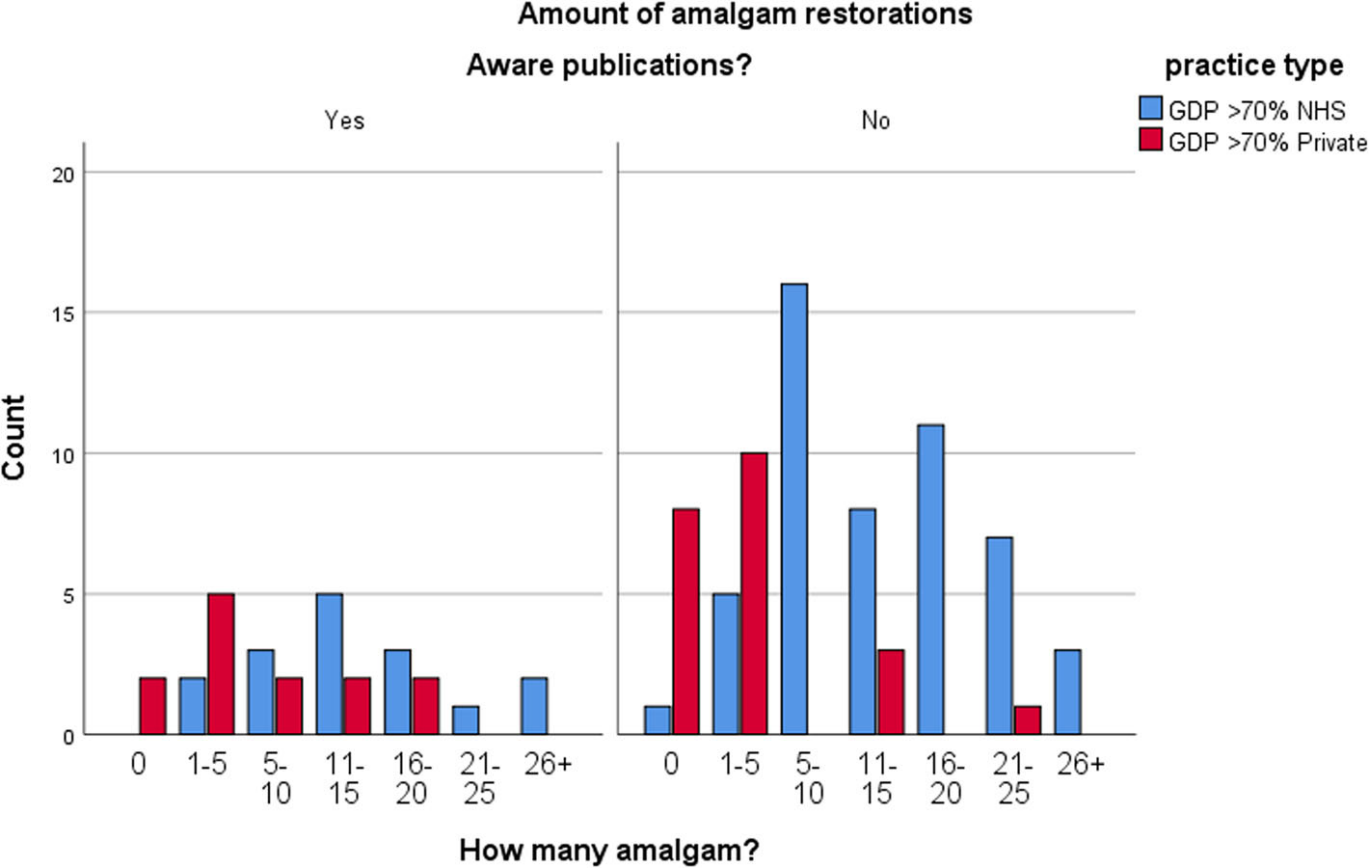
Stratified Amalgam usage by practice **-** Differences in amounts of amalgam restorations by NHS or private practitioners, stratified by the knowledge of amalgam phase-down

**Fig. 6:**
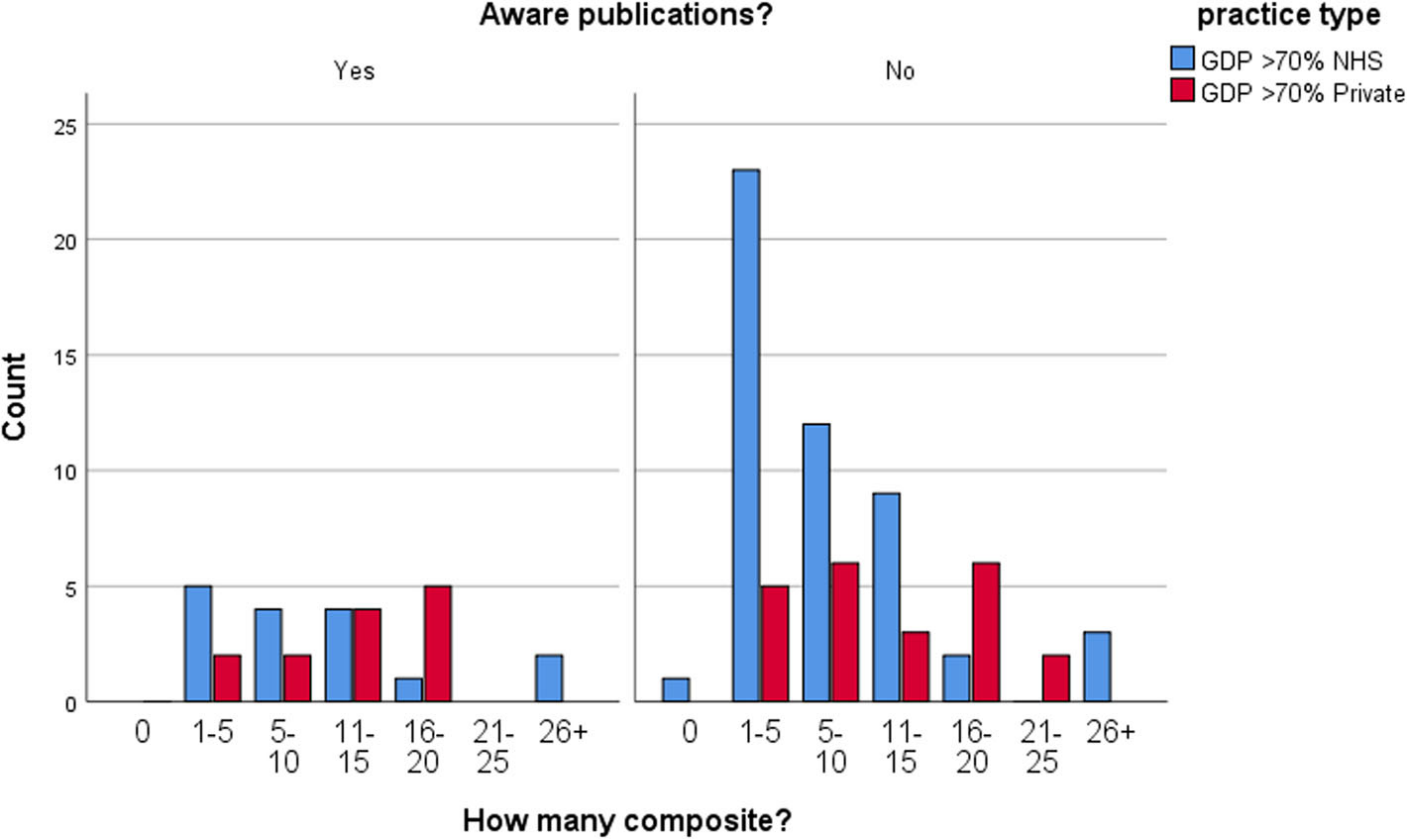
Stratified composite usage by practice **-** Differences in amounts of composite restorations by NHS or private practitioners, stratified by the knowledge of amalgam phase-downs

The survey of Yorkshire dentists showed that it was predominantly NHS practitioners who carried out a higher number of amalgam restorations on average with 60% (*n* = 40) placing significantly (*p* < 0.001) more than 10 amalgams per week compared to 22% (*n* = 8) of predominantly private practitioners (Fig. 3). 31% (*n* = 11) of private practitioners did not place any amalgams at all. NHS practitioners also reported placing posterior composites with 32% (*n* = 21) placing more than 5 composites per week compared with 56% (*n* = 20) of private practitioners (*p* = 0.002) placing the same (Fig. 4). Although significantly attenuated, these trends remained unchanged for number of amalgams and composites when we stratified according to an awareness of amalgam phase-down. For current usage of amalgam (*p* = 0.257 for those aware of a phase down versus *p* < 0.001 for those not aware) (Fig. 5) and composite (*p* = 0.173 for those aware of a phase down versus *p* = 0.016 for those not aware) (Fig. 6).

### Response rates

#### Quantitative phase

One hundred thirty-one usable replies were received for the Yorkshire dentists’ survey (giving response rate of 37%). Survey respondents were asked to identify how important a list of factors were in deciding their choice of restorative material (not important at all, neither important or unimportant, somewhat important, or very important). The most important factors in choosing a restorative material were the size/surfaces of the restoration, the ability to bond to tooth and the appearance of the material. 71% (*n* = 92) of respondents were not aware of any information regarding phasing-down amalgam. Respondents were asked to choose all factors that applied and rank then from ‘not important at all’ to ‘very important. Whilst they thought all factors were important in considering implementation of phase-down the most common responses were time, cost and problems with the NHS. When considering clinical photographs, NHS practitioners favoured the use of amalgam over composite and private practitioners the opposite. The main concerns over alternatives to amalgam were longevity (30%, *n* = 39), moisture control (26%, *n* = 33) and technique problems (22%, *n* = 29). Increased time for restorations (38%, *n* = 46) and the viability of business (27%, *n* = 32) were the most common concerns about effects on practice. 92% (*n* = 119) thought that amalgam was still a useful material. Although attenuated, the trends for these responses remained unchanged in our stratified analysis.

We also examined the effects of stratification on concerns for alternatives to amalgam. Overall, 102 (77.9%) practitioners were concerned about alternatives to amalgam. Of those practitioners aware of publications, 33 (86.8%) practitioners were concerned about alternatives; of those practitioners not aware of publications, 69 (75.0%) practitioners were concerned about alternatives. These results were not statistically significant (*p* = 0.14).

#### Qualitative phase

In total there were 23 participants. Three focus groups were conducted; 2 focus groups included GDPs working in a teaching hospital setting (*N* = 4 in each); 1 focus groups with hygienists and therapists working in a hospital teaching setting (*N* = 3).

Twelve telephone / face-to-face  interviews were conducted which comprised; Commissioners and policy makers (*N* = 3), Dental School Leads (*N* = 2), GDP NHS (*N* = 3), GDP Private (*N* = 2), GDP Community (*N* = 1) and Foundation dentist (*N* = 1).

### Impact of phase down

To meet objective 2: To explore the perceptions of GDPs, dental educators and dental commissioners on the impact of amalgam phase down on oral health inequalities.

The results from the free-text answers of the quantitative survey which were analysed using content analysis and the secondary analysis of the qualitative interviews revealed common themes relating to the impact of amalgam phase down on GDPs, patients and oral health service providers (NHS). The results of the analysis clustered around direct and indirect impact of amalgam phase down on oral health inequalities. Themes within direct impact were; cost, time and quality of care; with themes of indirect impact being; increased extractions, access problems and increased privatisation.

### Direct impact of amalgam phase down

#### Cost

The most predominate theme across participants was a consensus that the phase-down and ultimate removal of amalgam as a restorative material will have cost implications. Participants raised concerns over widening inequalities for those with worse oral health being more greatly affected.

*“moneywise especially in the area where I am working I don’t think people are going to afford to pay for it, we are very rare doing a composite especially if it’s on NHS we use on posterior teeth we do it for £50 or £60 they say no just put an amalgam on. If they have to pay for it I’m sure they-it’s already a deprived areas and high need in dentistry and their oral hygiene is-so I think this is going to create problems … .” (GDP NHS) (Interview)*

With concern over the need for more “*transparency”* of “*potential costs*” and fears that “*nobody has actually looked at the cost of this implementation*” (Commissioner Interview). Commissioners explaining that there is no money for such an increase in cost to the NHS “*so this is all going to have to be absorbed within the existing budget”*(Commissioner Interview). With a need for “*an evidence base on exactly what the choices are going to be when amalgam actually goes, nobody has actually looked at the cost of this implementation both in financial terms and also in time … .it’s going to take twice as long and cost three times as much”(*Commissioner Interview). Findings from the survey of Yorkshire dentists also raised concerns over the “*NHS - big issue of funding – takes much longer to place and if you are going to do things properly equipment is much more costly i.e. rubber dam and sectional matrix systems” (*Dentists’ Survey).

### Time

Another direct impact highlighted by participants was the increased time it would take to place amalgam alternatives with amalgam described as “*less time consuming*” (GDP NHS Focus Group).

*“I think the biggest issue I think is time for Dentists. The alternatives tend to be slower and less reliable. So unless you’ve got all time in the world to spend over it, you know, it’s difficult “(Dental School Lead Interview)’*

This is very closely related to costs as mentioned previously in terms of appointment/chair side time with alternatives “*a lot longer than putting amalgam in*” resulting in composites “*not done well because they can’t get through the composites with the time they were allocated doing it correctly”(GDP Hospital Focus Group).* With the time allocations and targets described as “*physically impossible*” to meet with the increased time it takes for alternative materials. A sentiment directly mirrored in the responses to the survey data adding that; *“time taken to place, polish etc under rubber dam not feasible with NHS funding” (Dentists Survey).* With all participants reporting that such time constraints would have a direct impact upon the quality of placing alternative restorations.

### Quality

Participants described a potential direct impact of reduced quality of fillings due to time constraints mentioned above but there were also considerable concerns regarding the quality of amalgam alternatives.

*“I think you will be looking at more repeats … … I’ve got a composite I’ve now re-done three times. They’ve just got a shorter lifespan, it’s a softer materials, you get a lot more leakage around the sides” (Foundation Dentist Interview).*

*“And that’s the difficulty there’s a lot of anecdote but not an awful lot of evidence about longevity of current composite restorations compared to ones which were placed 10-15 years ago” (Dental School Lead Interview)*

“*Longevity of restoration*” and “*frequency of replacement*” were also concerns raised by the dentists completing the survey and especially with wear “*If GIC is used it does wear down more quickly” (GDP Survey)* with claims that *“glass ionomer materials do not last long enough to be a long-term alternative for posterior restorations”(GDP Survey).* Both within the interviewed participants and those GDPs who responded to the survey of Yorkshire dentists, highlighted a number of concerns regarding composite restorations which would be the alternative of choice to replace amalgams. Moisture control was mentioned as an issue that would affect quality of restoration and was described as “*challenging” (GDP Interview)* compared to amalgam as *“Composite is very difficult in areas of poor moisture control” (GDP Survey).* Technique problems more generally were also mentioned by all participants as *“Amalgam is easy to use* “(GDP Survey) and “*best material*”(GDP Focus Group) whereas” *Composites are very technique sensitive”(GDP Survey)*

*“I worry that some bulk fill materials could be rushed when placed and not used under correct restricted moisture conditions/bonding. I feel the incremental build up using composite is probably the only ‘fail safe and predictable material” (GDP Survey).*

There were also some concerns raised over the safety and potential toxicity of alternatives with concerns raised over *“Bis-GMA and cancer”*(GDP Survey) *“Pulpal incompatibility with composites, biocompatibility of nano technology”*(GDP Survey).

*“We don’t know what impact bonding agents etc have on health”(GDP Survey)*

*“ … composites but what are they dissolving down into? You put a composite in 20 years down the line there’s a lot less of it then when you put it in, where’s it gone? … and what damage is that doing? (GDP NHS Interview)*

### Indirect impact of amalgam phase down

#### Increased extractions

Dentists and dental school leads expressed concern that there may be some indirect consequences of the reduction in amalgam use that could lead in the longer term to worsening inequalities. Meaning that for some due to the direct impact of cost, from both the dentist and patient perspective, would lead to there being more extractions.

*“Yes, I suspect in the more deprived areas without amalgam there’d probably be more teeth extracted as the alternative as the quicker, more reliable, alternative … ” (Dental School Lead Interview)*

*“if costs go up it might change patients decisions because some patients are driven by cost and I’ll say yes I’ll have the tooth out instead” (GDP NHS Interview)*

Yorkshire dentists also commented in their survey responses that there would not only be more extractions but also crowns *“Pts, especially patients who could not afford crowns may lose more teeth as a result”.* As a result of concerns highlighted in the theme of *‘quality’* mentioned previously this could also lead indirectly to a greater number of extractions with *“Endo and extractions will go through the roof due to substandard placements”* (GDP Survey).

*“It would limit my ability to offer effective restoration of teeth in areas where access and moisture control are difficult. More extractions likely”(GDP Survey)*

*“there’s going to be more teeth deemed as unrestorable and ultimately will need extracting then you get to the stage where if you’ve got a really deep cavity very subgingival there’s a fine line, does it need to stay does it need to go?” (GDP Hospital Focus group)*

### Access problems

Commissioners and dentists felt that the additional time taken to place composites would result in dentists seeing fewer patients therefore having an indirect impact upon access to the dentist, causing problems particularly in high need areas.

*“and the time that you’ve spent placing that composite is time you cannot then offer to your other NHS patients, and in a population where they are increasingly asking us to see more and more patients how are we going to find to time to see those more patients and place all their composites you know we’re going to have to have a contract where we’re required to see more patients really” (GDP hospital Focus Group)*

*“the affect it’s going to have on access. So people … you know if it cuts down by 1/16*^*th*^ *the amount of people are going to see a Dentist then that’s the amount of time, the extra time it takes for us to actually do that particular procedure. It’s just they will see less people and then oral health will probably start to go down again” (Commissioner Interview)*

*“You’ve got 10 minutes to do an MOD restoration. You can do that with an amalgam, you can’t do that with a composite. You can but it’s not going to be a great one. If I did it, it would be terrible” (GDP NHS Interview)*

#### Privatisation

Dentists frequently commented about the viability of the dental business under the current NHS healthcare system and this was also echoed by dental school leads. That the ultimate combination of increase *costs* and lack of *time* would lead to greater privatisation of current NHS practice, ultimately worsening inequalities by creating barriers of cost, access and availability.

*“I am guessing that some of these composites that we see may well have not done well because they can’t get through the composites with the time that they are allocated doing it correctly, how would i, i have been in some NHS jobs where you are working to targets, how are you supposed to physically it’s impossible” (GDP hospital Interview)*

That working without amalgam and still within the boundaries of NHS would render practice ‘*impossible*’ forcing poorer quality of restoration and “*more repeats*” (Foundation dentist Interview) or even “*3 UDAs for 1 hours work will leave practices bankrupt*” (GDP Survey).

*“Likely to become entirely private. Definitely not feasible with current NHS contract”(GDP Survey)*

Those that have already been operating within the private sector and also been amalgam free “*for a while they get by, by using the alternative materials but charging the right amount of money to spend the right amount of time on it” (dental school lead Interview).* Showing that the impact for those that are able to pass on the financial burden to their patients don’t encounter many difficulties in reducing amalgam use.

*“I just decided just not to use it one day and stopped buying any and moved on from there. It’s a bit like when I gave up working in the NHS, I just went cold turkey and said right I don’t have an NHS contract so I can’t give NHS treatment for you and most patients accept that and the same would go for if you change material, well I don’t have any amalgam but we have this what would you like me to do and most people will say yes carry on” (GDP PRIVATE)*

## Discussion

To our knowledge this is the first in-depth investigation of the perceived consequences of implementing a complete phase out of dental amalgam. Whilst the quantitative data showed that there has been a reduction in amalgam use, it still remains a commonly placed material and the material of choice for large restorations in posterior teeth. Worryingly, our data show that a complete phase down on amalgam will result in a widening or oral health inequalities with those not able to afford amalgam alternatives being subjected to increased extractions, reduced access to dental services and poor quality of treatment. The former was primarily due to increased time and costs of placing alternative materials and the latter related to challenges of placing amalgam alternatives particularly those pertinent to moisture control and long term durability of alternatives. These finding are in agreement with the post-Norwegian evaluation of amalgam phase out [3, 10] which showed that larger fillings using amalgam alternatives were more time consuming, less durable and reported to cause allergic reactions in the mouths of patients and on hands of personnel [3, 10]. Most of the costs related to the phase out were due to the increased time spent on dental clinics when using alternatives and due to more frequent attendance to replace fillings [3]. Whilst the most popular alternative was composite [10] thus demonstrating acceptance, its longevity was still questionable with only 45% of dentists reporting longevity of > 10 years for composites compared with 71% who thought longevity would be longer if the material was amalgam [10].

The findings of both the post-Norwegian evaluation and those of our study provide strong evidence of the adverse impact of an amalgam phase out on costs and provision of oral health services. Whilst in Norway, the additional costs of providing amalgam alternatives were absorbed by the adult consumer [3], it is clear from our findings that a phase down will impose a significant financial drain on oral health services that are paid for by the state with both dentists and commissioners warning of access problems and increased costs. Given that people from low socioeconomic areas have higher rates of untreated dental disease namely caries (tooth decay), periodontal (gum) disease, tooth loss, oral cancer and oral pain than their affluent counterparts [11–14], increased costs of amalgam alternatives will widen oral health inequalities by the inability of the poor to afford such alternatives. On a global level, the impact on oral health inequalities is likely to be worse in low and middle income countries where there are disturbingly high levels of oral health inequality within and between these countries [15]. This will be further amplified by limited access to oral health services following a complete phase down of amalgam given that people living in deprived areas are already less likely to access dental services than their more affluent counterparts [16–18].

Particular strengths of our study included the mixed methods design that allowed us to sample purposively and triangulate the views of commissioners of state run oral health services, dentists working both in the state and private sector and dental educators to gain an in-depth understanding of the impact of amalgam phase out on the major stakeholders involved in amalgam use. The use of two researchers who independently coded and analysed the data also added to methodological rigour. The number of participants was sufficient to allow thematic saturation and to explore deviant cases amongst groups and revealed common themes relating to the problems of a complete amalgam phase down. The qualitative findings further corroborated the findings from a survey of randomly selected dentists working in the state and private sectors. The usage of amalgam was estimated using national data of the state run service over a 10 year period and the survey of dentists was used to assess differences between the state run and private sector. This showed a much higher usage of amalgam in the state run National Health Service. Demand for dental care increases through greater coverage by insurance and state run services which provide a policy tool for tackling oral health inequalities [19]. The dependence of such services on dental amalgam further suggests a widening of oral health inequalities in countries that are reliant on such systems for provision of oral health care and which are commonly accessed by lower socio-economic groups who have higher caries rates.

However, there are come limitations that need to be considered. First, we conducted a descriptive analysis of the results from the 131 questionnaires from the Yorkshire survey which provided valuable information about the opinions of dentists in West Yorkshire. In particular we were able to assess general trends for current practice, choice of materials and concerns over amalgam alternatives. However, the low response rate of the survey could have led to selection bias. In addition, survey questions such as 5, 6, 7 (Additional file 1) may have been unbalanced and pushed towards factors being considered important as these questions were closed and not open-ended. To mitigate this we conducted the qualitative study which asked open ended questions and the results show similar responses which corroborate the findings from the survey. In addition, our findings were also in agreement with evidence from post-amalgam ban Norwegian experiences [3] and cost data for amalgam versus alternatives [4]. Second, the survey could have suffered from misclassification bias particularly in relation to knowledge of an amalgam phase down. To account for potential differences in response with regard to awareness of an amalgam phase down, we stratified the findings from the postal survey according to responses to this question (question 6 Additional file 1). This showed that an awareness of amalgam phase-down did not affect the observed trends in the descriptive data although numbers were significantly attenuated. Third, interviewer bias also needs careful consideration in both the quantitative and qualitative studies. Given that the survey of dentists was posted to a random sample of dentists, interviewer bias is unlikely to have been an issue in the postal survey. For the qualitative interviews we ensured that these were conducted by two trained interviewers using a structured topic guide (Additional file 2) which minimised interviewer bias. Further, use of snowballing for selection of dental practitioners could have resulted in selection bias for the qualitative study. To mitigate effects of this, we interviewed divergent cases, (practitioners who had completely phased out the use of amalgam) and triangulated views from other stakeholders (dental educators and commissioners).

The findings of our study are likely to have implications for future policies and decision making in amalgam phase out. We have shown that a complete phase out of amalgam is likely to adversely impact reimbursement of health professionals which in turn will affect access to care as well as the extent and quality of care. The findings of the post-Norwegian evaluation of amalgam phase-out [3] suggest that additional costs of a phase down are likely to be absorbed by patients which will in turn widen oral health inequalities. Future research needs to extend the findings of our study to interrogate patient views on amalgam phase down based on stakeholder perceptions identified in our study.

## Conclusions

Given that incentives for both patients and dentists need to be taken into account when designing health policies to tackle inequalities [20, 21] and that both these will be adversely affected by a complete phase out of amalgam, policies geared towards a complete ban on amalgam need to carefully consider their likely impact on widening oral health inequalities. Our data suggest that a complete phase out is not currently feasible unless appropriate measures are in place to ensure cheaper, long-lasting and easy to use alternatives are available and can be readily adopted by primary care oral health providers. These findings provide a timely opportunity to explore the implications for reforming dental care.

## Supplementary information

**Additional file 1.** Questionnaire for Yorkshire Survey of Dentists.

**Additional file 2.** Topic guide for qualitative interviews.

## Data Availability

The data that support the findings of this study are available from Information Services Division, NHS National Services Scotland but restrictions apply to the availability of these data, which were used under permission for the current study, and so are not publicly available. Data are however available from the authors upon reasonable request and with permission of Information Services Division, NHS National Services Scotland.

## Abbreviations

BDA: British Dental Association
FDI: World Dental Federation
GDP: General Dental Practitioner
GDS: General Dental Services
IADR: International Association of Dental Research
MIDAS: Management Information & Dental Accounting System
NHS: National Health Service
SDR: Statement of Dental Remuneration
UDAs: Units of Dental Activity
UK: United Kingdom
