# Supplementary material for: Assessing the perceived impact of post Minamata amalgam phase down on oral health inequalities: a mixed-methods investigation
Source: BMC Health Serv Res. 2019 Dec 21;19:985. doi: 10.1186/s12913-019-4835-1 (PMC6925872; doi:10.1186/s12913-019-4835-1)
Supplement: Supplementary file 1 — Additional file 1. Questionnaire for Yorkshire Survey of Dentists. [file 12913_2019_4835_MOESM1_ESM.docx]

Questionnaire for Survey of Yorkshire Dentists

Question 1:

Year of graduation:

🞏 Before 1980

🞏 1981-1985

🞏 1986-1990

🞏 1991-1995

🞏 1996-2000

🞏 2001-2005

🞏 2006-2010

🞏 2010 – present

Question 2:

Type of practice (tick all that apply):

🞏 General practitioner – predominately (>70%) NHS

🞏 General practitioner – mixed

🞏 General practitioner – predominately (>70%) private

🞏 Specialist practitioner

Speciality ………………………………………………………………………………………………

🞏 Hospital practitioner

Speciality ………………………………………………………………………………………………

🞏 Community practitioner

Question 3:

On average, how many hours of clinical duties do you work per week?

🞏 < 8

🞏 9 – 16

🞏 17 – 24

🞏 25 – 32

🞏 33 – 40

🞏 41 – 48

🞏 48 +

Question 4:

How many amalgam/composite/alternative posterior restorations do you currently place per week on average?

Amalgam Composite Alternative

0 🞏 🞏 🞏

1-5 🞏 🞏 🞏

5-10 🞏 🞏 🞏

11-15 🞏 🞏 🞏

16-20 🞏 🞏 🞏

21-25 🞏 🞏 🞏

26 + 🞏 🞏 🞏

Question 5:

Looking at the following factors, how important are they in your choice of restorative material?

|  | Not important at all | Neither important or unimportant | Somewhat important | Very important |
| --- | --- | --- | --- | --- |
| Cost of material |  |  |  |  |
| Ease of placement |  |  |  |  |
| Familiarity of material |  |  |  |  |
| Ability to bond to tooth |  |  |  |  |
| Appearance |  |  |  |  |
| Size/surfaces of restoration |  |  |  |  |
| Patient finances |  |  |  |  |
| Evidence base |  |  |  |  |

Question 6:

Are you aware of any publications or information regarding a reduction in the amount of amalgam that is used in the UK?

🞏 Yes 🞏 No

If yes, where did you see this?

|  |
| --- |

Question 7:

What do you think are the most important factors that will affect the ability of the profession to implement a phase-down in amalgam?

|  | Not important at all | Neither important or unimportant | Somewhat important | Very important |
| --- | --- | --- | --- | --- |
| Cost of materials |  |  |  |  |
| Time taken for placement |  |  |  |  |
| Funding from central source (NHS) |  |  |  |  |
| Patient ability to pay |  |  |  |  |
| Dentist education and training |  |  |  |  |
| Patient education |  |  |  |  |
| Viable alternative materials |  |  |  |  |
| Other please state below: |  |  |  |  |

If you would like to explain your answer please do so below below:

|  |
| --- |

Question 8:

In your normal practice what would you restore this tooth with? Please rank 1-3 in order of your personal preference, the patient has no preference and is happy for you to advise what you consider to be the best restoration.


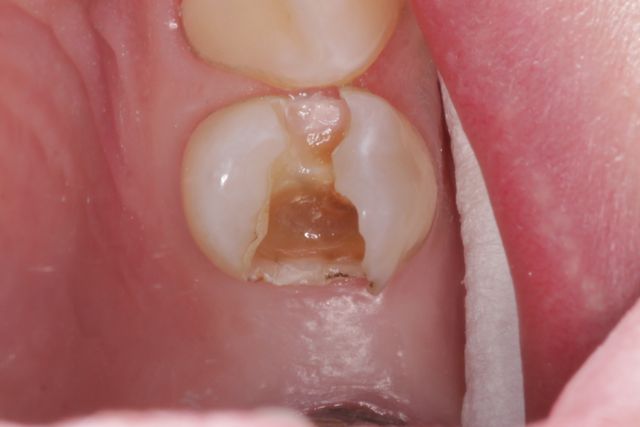


UR4 MOD

🞏 Amalgam

🞏 Composite

🞏 Resin-modified GIC

🞏 GIC

🞏 Metal inlay/onlay

🞏 Porcelain inlay/onlay

🞏 Full coverage crown

🞏 Other – ………………………………


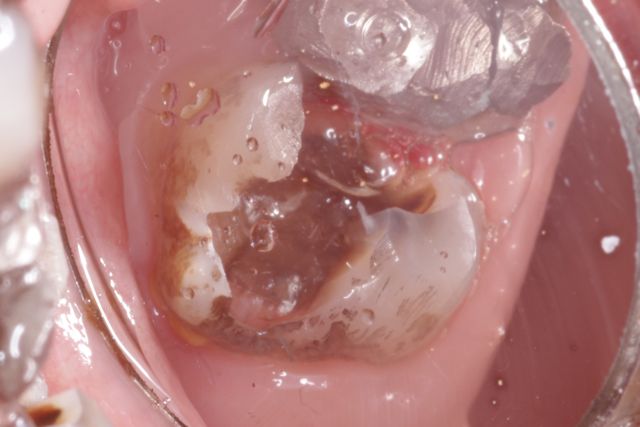


UL7 MO (extends subgingivally)

🞏 Amalgam

🞏 Composite

🞏 Resin-modified GIC

🞏 GIC

🞏 Metal inlay/onlay

🞏 Porcelain inlay/onlay

🞏 Full coverage crown

🞏 Other – ………………………………


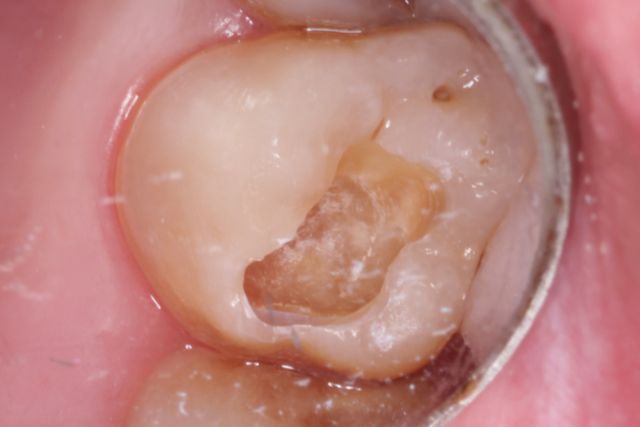


UR6 O - large

🞏 Amalgam

🞏 Composite

🞏 Resin-modified GIC

🞏 GIC

🞏 Metal inlay/onlay

🞏 Porcelain inlay/onlay

🞏 Full coverage crown

🞏 Other – ………………………………


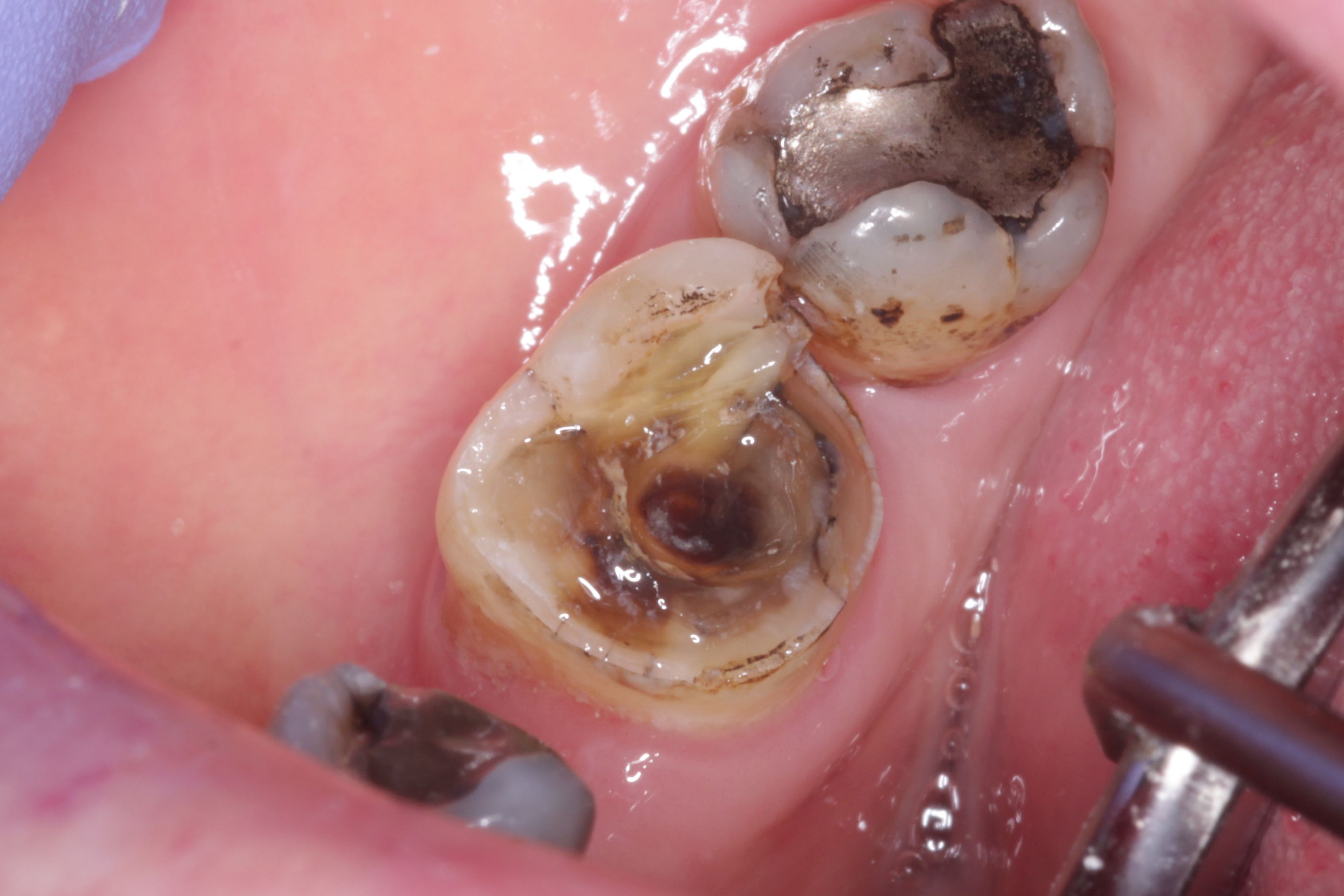


LR6 - MODL

🞏 Amalgam

🞏 Composite

🞏 Resin-modified GIC

🞏 GIC

🞏 Metal inlay/onlay

🞏 Porcelain inlay/onlay

🞏 Full coverage crown

🞏 Other – ……………………………

Question 9:

When considering alternative restorative materials to amalgam, do you have any concerns over the materials that could be used?

🞏 Yes 🞏 No

If Yes then please explain why below:

|  |
| --- |

Question 10:

a) If you were not able to use amalgam as a material how would this affect your practice?

|  |
| --- |

b) Do you think that amalgam is still a useful material for restoring posterior teeth?

🞏 Yes 🞏 No 🞏 Unsure

Please explain your answer below:

|  |
| --- |

If you have any further comments that you would like to make on this subject then please note them below.

|  |
| --- |

Thank you for taking the time to complete this questionnaire.
