# Supplementary material for: Assessing the perceived impact of post Minamata amalgam phase down on oral health inequalities: a mixed-methods investigation
Source: BMC Health Serv Res. 2019 Dec 21;19:985. doi: 10.1186/s12913-019-4835-1 (PMC6925872; doi:10.1186/s12913-019-4835-1)
Supplement: Supplementary file 2 — Additional file 2. Topic guide for qualitative interviews. [file 12913_2019_4835_MOESM2_ESM.doc]

# TOPIC GUIDE

# Amalgam Phase Down – Understanding the implications for dentistry and dental education.

The semi-structured interview will be developed in parallel with the inductive analysis and focus groups. The following topics will be covered from the outset, examples of questions are provided of the types of prompts that will be used.

GDP guide:

Experience of working with amalgam

- What is your current role?
- Tell me about your experience of working with amalgam. Now? In previous roles?
- What proportion of patients that you see have amalgam restorations?
- How appropriate do you think it is for you to place alternative restorations?
- How do you find working with alternative restorations? Challenging? Frustrating? Interesting?

Views on amalgam phase out

- Are you aware of amalgam phase out? Minimata agreement.
- Are you prepared for a complete eventual ban on amalgam use and are you reducing the use of amalgam in your clinical practice?
- What barriers do you perceive in the phase out of amalgam
- How do you think it will impact your clinical practice?
- How do you think it will impact patients.

Dental school leads:

- Are you aware of amalgam phase out? Minimata agreement.
- Are you prepared for a complete eventual ban on amalgam use and are you implementing this in clinical teaching
- What proportion of amalgam restorations do students provide as part of their requirements?
- What barriers do you perceive in the phase out of amalgam
- How do you think it will impact undergraduate teaching?

Commissioner guide

- Are you aware of amalgam phase out? Minimata agreement.
- Do you think the current NHS and future planned NHS contracts are prepared for amalgam phase down?
- What barriers do you perceive in the phase out of amalgam
- What materials will you expect to commission as alternatives to amalgam on the NHS?
